# Spatial Omics Technologies in Glioblastoma Research: Principles, Applications, and Best Practices

**DOI:** 10.3390/genes17070822

**Published:** 2026-07-18

**Authors:** Maxime Vanmechelen, Chiara Caprioli, Paul M. Clement, Ann Hoeben, Frederik De Smet

**Affiliations:** 1 Laboratory for Precision Cancer Medicine, Translational Cell and Tissue Research Unit, Department of Imaging and Pathology, KU Leuven, 3000 Leuven, Belgium; 2 Leuven Institute for Single-Cell Omics (LISCO), KU Leuven, 3000 Leuven, Belgium; 3 Leuven Cancer Institute (LKI), KU Leuven, 3000 Leuven, Belgium; 4 Department of Medical Oncology, University Hospitals Leuven, 3000 Leuven, Belgium; 5 Department of Medical Oncology, GROW—Research Institute for Oncology and Reproduction, Maastricht University Medical Center (MUMC+), 6229 Maastricht, The Netherlands

**Keywords:** glioblastoma, spatial omics, tumor microenvironment, spatial ecosystems

## Abstract

Background/Objectives: Glioblastoma (GBM) remains the most aggressive primary brain tumor in adults, characterized by inevitable recurrence, extensive inter-and intratumoral heterogeneity, and resistance to current therapies. A defining feature of GBM is the dynamic interplay between malignant cells and a diverse tumor microenvironment (TME), which together drive disease progression, therapeutic adaptation, and relapse. Understanding these complex cellular ecosystems has therefore become a major focus of glioblastoma research. Recent advances in spatial omics technologies have transformed our ability to investigate GBM biology directly within intact tissue architectures. Over the past five years, an expanding array of spatial transcriptomic, proteomic, and multi-omic platforms has enabled high-dimensional characterization of cellular states, cell–cell interactions, and tissue niches while preserving spatial context. These approaches have generated unprecedented insights into tumor organization, cellular plasticity, immune landscapes, vascular niches, and treatment-induced ecosystem remodeling. Methods: In this review, we provide an overview of spatial omics applications in glioblastoma research so far. Results: We summarize the technologies employed, the types and numbers of patient samples analyzed, and the major biological and clinical insights generated. We compare the strengths and limitations of different spatial platforms, discuss key considerations for study design and data interpretation, and highlight emerging trends in multimodal and longitudinal analyses. Conclusions: By integrating both technological and biological perspectives, this review serves as a practical resource for researchers seeking to implement spatial omics approaches in glioblastoma studies and to advance precision neuro-oncology.

## 1. Introduction

Glioblastoma (GBM) is the most aggressive primary brain tumor in adults [1,2]. Despite intensive multimodal treatment consisting of maximal safe surgical resection followed by radiotherapy and temozolomide chemotherapy, recurrence is nearly inevitable with an overall survival that rarely exceeds beyond 2 years [3]. The aggressive clinical behavior of GBM and its limited, often transient, response to therapy can largely be attributed to its profound inter- and intratumoral heterogeneity and the ability of the tumor cells to adapt to perturbations. Importantly, rather than representing a homogeneous tumor mass, GBM is increasingly recognized as a complex, spatially organized ecosystem composed of diverse malignant cell populations embedded within a dynamic tumor microenvironment (TME) [4,5,6,7,8]. The TME itself exhibits remarkable spatial heterogeneity and is organized into distinct cellular and structural niches that differ in cellular composition, metabolic activity, vascular architecture, immune infiltration, and interaction with the surrounding brain parenchyma. These niches are shaped by local environmental factors, including oxygen availability, nutrient gradients, and tissue architecture, resulting in regional neighborhoods with unique biological properties. Within this ecosystem, cell–cell and cell–matrix interactions collectively govern tumor growth, invasion, immune evasion, and therapeutic resistance, resulting in niches that continuously evolve during disease progression and in response to treatment [4,5,9,10,11].

Understanding GBM therefore requires moving beyond a purely tumor cell-centric perspective and embracing the spatial organization and multicellular framework. However, capturing these complex ecosystem dynamics has proven challenging. Traditional bulk transcriptomic approaches provide valuable molecular information but average signals across millions of cells, thereby obscuring regional heterogeneity, neighborhood-specific biology, and localized therapeutic vulnerabilities. Single-cell technologies offer unprecedented resolution of cellular diversity and state transitions but require tissue dissociation, resulting in the loss of spatial context and the physical relationships that govern cellular behavior within intact tissue [12,13]. Consequently, neither approach alone can fully explain how cellular states, microenvironmental niches, and cell–cell interactions cooperate to drive disease progression.

Spatial omics technologies have therefore emerged as a powerful solution to this challenge by enabling high-dimensional molecular profiling while preserving tissue architecture. In this review, we provide an overview of the spatial omics technologies that have been applied to glioblastoma research, examining how these platforms have been used to interrogate tumor biology, how patient cohorts and tissue specimens have been selected and characterized, and to what extent spatial datasets have been integrated with clinical and molecular information (Table 1). This analysis reveals clear trends in technology adoption, cohort design, tissue sampling strategies, and biological discovery. By synthesizing these developments, we identify emerging best practices, highlight current limitations, and outline future opportunities.

## 2. Materials and Methods

A literature search was conducted in February 2026, focusing on research articles and review papers containing spatial omics technologies on glioblastoma patient samples. An online literature search in Medline was performed for relevant articles, using the search terms (1) ‘glioblastoma’ + ‘spatial transcriptomics’, (2) ‘glioblastoma’ + ‘omics’ and (3) ‘glioblastoma’ + ‘single cell spatial technologies’ with publication date over the past 5 years. Articles on tumors other than IDHwt glioblastoma were excluded. We further expanded the search using the ‘related article’ function and by including references from the initial selection. During the preparation of this manuscript, the authors used FigureLabs (https://www.figurelabs.ai, accessed on 14 June 2026) for the purposes of preparing the graphical abstract. The initial layouts were vectorized and subsequently manually refined, labeled, and verified by the authors to ensure scientific accuracy.

## 3. Comparative Analysis of Published Spatial Omics Cohorts

To better characterize the current landscape of spatial omics in glioblastoma, we performed an elaborate comparison of landmark studies that integrated spatially resolved molecular profiling of human glioblastoma specimens (Table 1). Rather than focusing solely on the biological findings reported by each study, we extracted a standardized set of technical, biological and clinical parameters to assess how the field has evolved over time and to identify remaining opportunities for future research. This comparative framework allows studies employing different technologies and analytical approaches to be evaluated on common criteria, providing an overview of the strengths and limitations of existing glioblastoma spatial omics resources.

For each study, we recorded the clinical composition of the cohort, including the number of patients, the inclusion of newly diagnosed and/or recurrent tumors, and whether matched longitudinal samples were available. These parameters determine the extent to which datasets can capture inter-patient heterogeneity and tumour evolution during therapy. We additionally catalogued the molecular modalities incorporated into each study, including genomic sequencing, DNA methylation profiling, single-cell transcriptomics, spatial transcriptomics and multiplex immunohistochemistry. Because each modality interrogates a different layer of tumour biology, this assessment illustrates the increasing shift from single-technology studies towards integrated multi-omics approaches capable of linking genetic alterations, transcriptional plasticity, protein expression and spatial organization. To quantify the depth of molecular profiling, we extracted measures such as the number of tissue sections analyzed, the number of single cells profiled, the extent of spatial coverage, and the dimensionality of multiplexed assays (for example, the number of protein markers or transcriptomic features measured). These parameters reflect the analytical resolution with which tumor ecosystems can be reconstructed and determine the statistical power for identifying conserved cellular states and spatial niches across patients.

Finally, we documented the availability of complementary clinical and pathological annotations, including treatment history, MGMT methylation status, molecular subtype, survival data and other clinicopathological variables. Such metadata are essential for translating descriptive spatial atlases into clinically meaningful resources by enabling associations between spatial ecosystem architecture and patient outcome or therapeutic response. Collectively, these parameters capture the three defining dimensions of modern glioblastoma spatial omics studies: clinical breadth, molecular depth and multimodal integration. Comparing published cohorts across these dimensions reveals not only the remarkable technological progress achieved over the past decade, but also the persistent challenges that must be addressed to generate reference atlases capable of supporting biological discovery, biomarker development and precision oncology.

## 4. Evolution of Single-Cell and Spatial Omics in Glioblastoma

Although spatial omics technologies have only recently been applied to GBM, several remarkably consistent themes have emerged across studies despite substantial differences in platforms, cohort composition, analytical approaches, and molecular modalities. The foundations for this conceptual framework were established through single-cell transcriptomic studies, which fundamentally reshaped our understanding of GBM heterogeneity. Landmark analyses demonstrated that malignant cells are not randomly heterogeneous but instead occupy a number of transcriptional states that recapitulate developmental hierarchies of the normal brain. Initial studies identified four major cellular programs (i.e., neural progenitor-like (NPC-like), oligodendrocyte progenitor-like (OPC-like), astrocyte-like (AC-like), and mesenchymal-like (MES-like) states), which have subsequently been validated across numerous independent cohorts and experimental platforms [16,18,25,29]. Importantly, these programs do not represent fixed cellular identities but rather dynamic and reversible states that can coexist within the same tumor and even within individual genetic clones. More recently, this framework was expanded by identifying additional populations, including glial progenitor cell-like (GPC-like), neuron-like (NL-like), and cilia-like (CL-like) cells, revealing that the original four-state model represented a simplified view of a far more continuous landscape of cellular identities [16,19,29]. Together, these studies established cellular plasticity as a defining feature of GBM biology and challenged the traditional concept of discrete molecular subtypes.

Despite these advances, single-cell approaches provided only a partial view of tumor organization. Although they successfully identified the cellular components of the GBM ecosystem, they could not determine where these populations were located within intact tissues or how they interacted with one another. The introduction of spatial transcriptomic and proteomic technologies therefore represented a critical conceptual advance by enabling investigators to move beyond cellular inventories and begin reconstructing the ecological organization of glioblastoma. Moreover, as spatial omics technologies evolved towards higher resolution while capturing gradually more biomolecules (from one to several thousand), the depth and granularity of biological insights have increased accordingly. Early studies relied largely on region of interest (ROI)-based approaches, such as GeoMx Digital Spatial Profiling, which enabled comparisons between predefined tumor compartments but provided limited information regarding cell–cell interactions (Table 2). The introduction of spatial transcriptomic platforms such as Visium subsequently enabled transcriptome-wide profiling across larger tissue sections albeit at lower resolution (average of 10–20 cells/spot), allowing investigators to reconstruct regional ecosystem patterns and identify spatially restricted signaling programs. Subsequently, single-cell and subcellular resolution technologies, including Xenium, CosMx, MERFISH-derived platforms [12], and multiplexed immunofluorescence approaches (MILAN, CycIF, among others [30], have further transformed the field (Table 2). Although initially only capable of capturing lower numbers of markers (10–100), these methods have shifted the focus from regional biology toward direct investigation of cellular neighborhoods and physical interactions. Rather than simply identifying which cell types coexist within a tumor, researchers could now determine which cells are adjacent to one another and how these interactions change during disease progression or treatment. Further advances in transcriptomic technologies now enable near whole-transcriptome measurements at single-cell and subcellular resolution [31], while multiplexed proteomic platforms have expanded from a handful of markers to the simultaneous detection of several hundred proteins within intact tissue sections. Importantly, the most significant conceptual advance may not be the increase in molecular depth alone, but rather the ability to integrate multiple modalities within a single spatial framework. Early multimodal studies typically relied on measurements performed on adjacent tissue sections, requiring computational alignment to infer relationships between molecular layers. In contrast, emerging spatial multi-omic technologies now permit the simultaneous quantification of multiple biological modalities, including RNA, protein, chromatin accessibility, genomic alterations, and cellular morphology, directly within the same tissue section and within the same individual cells. This transition has fundamentally altered the scope of spatial biology by enabling direct integration of complementary molecular information without the uncertainty introduced by tissue heterogeneity between serial sections.

## 5. Landmark Spatial Omics Discoveries in Glioblastoma

While technological innovation has driven the rapid expansion of spatial omics, the true impact of these approaches lies in the biological insights they have generated. Over the past several years, spatial analyses have fundamentally transformed our understanding of glioblastoma organization, revealing that tumors are composed of recurrent ecosystem structures rather than randomly distributed cell populations. These studies collectively demonstrate that spatial context is a major determinant of tumor behavior, therapeutic response, and clinical outcome. Rather than merely identifying individual cell types, spatial omics has enabled investigators to understand how malignant, immune, vascular, and neural populations interact within distinct microenvironmental niches. Several recurring biological themes have emerged across independent studies and technologies, suggesting that common organizational principles underlie glioblastoma progression despite substantial inter-patient heterogeneity.

### 5.1. Spatial Organization of Malignant Cell States

While single-cell profiling identified a multitude of cell states, their spatial organization remained largely unresolved. Using spatial omics analyses, we have learnt that the developmental tumoral states are not randomly distributed throughout tumors but preferentially localize to specific microenvironmental niches. OPC-like and NPC-like populations frequently reside in perivascular regions characterized by increased nutrient availability and vascular support, whereas MES-like populations are enriched in hypoxic, inflammatory, and immune-rich areas. AC-like states often occupy intermediate regions and may represent adaptive responses to local environmental conditions. Importantly, spatial analyses demonstrated that transitions between these states are strongly influenced by local microenvironmental signals. Rather than representing stable cell identities, developmental programs appear highly plastic and responsive to hypoxia, inflammation, metabolic stress, and therapeutic interventions. This observation has shifted the field away from static classification systems toward dynamic ecosystem models in which malignant cell states continuously interact with their surrounding microenvironment. Large-scale integration efforts such as Gbmap [17] further demonstrated that developmental states organize into recurring spatial ecosystems that are conserved across patients. Despite profound genomic heterogeneity, these ecosystem structures repeatedly emerge, suggesting that common environmental constraints shape glioblastoma architecture.

### 5.2. Perivascular Ecosystems and Stem Cell Niches

Among the most consistently observed spatial structures in glioblastoma are perivascular niches. Historically, these regions were recognized as reservoirs of glioma stem-like cells, but spatial omics has considerably refined our understanding of their complexity and functional importance. Spatial transcriptomic studies have demonstrated that perivascular regions contain distinct populations of OPC-like and NPC-like malignant cells enriched for stemness-associated transcriptional programs [20,32]. These cells frequently exhibit elevated expression of developmental regulators, growth factor receptors, and signaling pathways associated with self-renewal and therapeutic resistance. The vascular compartment itself is far from passive. Endothelial cells actively communicate with neighboring malignant populations through signaling pathways involving Vascular Endothelial Growth Factor (VEGF), Platelet Derived Growth Factor (PDGF) and Notch [33,34]. These interactions contribute to the maintenance of stem-like states and may promote adaptation to therapeutic stress. Recent work by Ren and colleagues identified radial glial stem-like populations preferentially localized within specific vascular niches in both glioblastoma and diffuse midline glioma [35]. Functional studies demonstrated that these niche-associated populations possess unique vulnerabilities that may provide opportunities for therapeutic intervention. Importantly, not all vascular niches appear biologically equivalent. Emerging evidence suggests substantial heterogeneity among perivascular regions, with some supporting highly proliferative stem-like populations while others exhibit increased immune infiltration or vascular remodeling. Future studies will likely further subdivide these ecosystems into distinct functional subtypes with differential prognostic significance.

### 5.3. Hypoxic and Necrotic Niches as Engines of Tumor Evolution

Hypoxia has long been recognized as a hallmark of glioblastoma pathology. However, spatial omics technologies have revealed that hypoxic regions are not merely passive consequences of insufficient vascularization, but rather active organizers of tumor ecosystem structure. Spatial transcriptomic analyses consistently demonstrate enrichment of MES-like malignant populations in hypoxic and perinecrotic regions [7,17,20,23,36]. These cells exhibit activation of HIF-dependent pathways, metabolic reprogramming, extracellular matrix remodeling, and inflammatory signaling networks. Hypoxia also profoundly influences non-malignant populations, including macrophages, endothelial cells, and reactive astrocytes. The landmark study by Ravi et al. [20] demonstrated that environmentally stressed tumor regions undergo coordinated transcriptional adaptation involving both malignant and non-malignant cell populations. These findings established hypoxia as a central driver of tumor-host interdependence rather than solely a metabolic stressor, which was confirmed by Greenwald et al. [7]. More recently, GBmap identified multiple hypoxia-associated ecosystems that correlate strongly with adverse clinical outcomes [17]. Interestingly, highly organized hypoxic niches appeared to possess greater prognostic significance than hypoxia-related gene expression alone, suggesting that spatial architecture itself may carry biological and clinical information. Hypoxic niches also represent major centers of immune suppression. Spatial analyses consistently demonstrate enrichment of immunosuppressive macrophage populations, altered antigen presentation pathways, and reduced cytotoxic lymphocyte infiltration within these regions. Collectively, these observations position hypoxic ecosystems as major drivers of tumor progression, immune evasion, and therapeutic resistance.

### 5.4. Invasive and Neuron-Associated Niches

One of the greatest clinical challenges in glioblastoma is its diffuse infiltration into surrounding brain tissue. Despite aggressive surgical resection, infiltrative tumor cells remain behind and ultimately drive recurrence. Historically, these populations were difficult to study because of their low abundance and intimate intermingling with normal brain tissue. Spatial omics technologies have provided unprecedented insights into the biology of invasive tumor regions. Studies examining tumor–brain interfaces consistently demonstrate that infiltrating malignant cells differ substantially from cells located within the tumor core. These populations exhibit increased expression of developmental genes, neuronal interaction pathways, synaptic signaling components, and programs associated with migration and plasticity. Harwood and colleagues [37] recently demonstrated that infiltrative glioblastoma cells exhibit enhanced expression of Notch signaling components and synaptic interaction genes within invaded brain tissue. These findings support the growing concept that malignant cells actively exploit neuronal signaling networks to facilitate invasion and survival. A related breakthrough was the identification of oncostreams, highly organized multicellular fascicles composed of aligned spindle-shaped cells exhibiting mesenchymal characteristics. Spatial analyses by Comba and colleagues [38] demonstrated that oncostreams function as dynamic migration highways capable of coordinating collective cellular movement throughout the tumor. The identification of COL1A1 as a key regulator of oncostream organization further suggested that spatially organized invasion programs may be therapeutically targetable. Collectively, these studies indicate that invasion is not simply a property of individual cells but rather an ecosystem-level phenomenon involving coordinated interactions between malignant cells, neurons, glia, extracellular matrix components, and vascular structures.

### 5.5. Spatial Architecture of the Immune Microenvironment

Perhaps no aspect of glioblastoma biology has been more profoundly transformed by spatial omics than our understanding of the immune microenvironment. Traditional models frequently categorized macrophages as either M1 or M2 polarized populations. However, single-cell and spatial analyses have revealed a far more complex reality. Glioblastoma-associated macrophages exist as multiple transcriptionally and spatially distinct populations, including SPP1+, TREM2+, APOC1+, FOLR2+, and MARCO-associated states, each exhibiting unique distributions and functional characteristics [39]. High-dimensional imaging studies demonstrated that immune cells organize into distinct spatial neighborhoods rather than being randomly dispersed throughout the tumor. Certain macrophage populations preferentially localize to hypoxic regions, whereas others accumulate around blood vessels or invasive fronts. These spatial distributions strongly influence local cytokine signaling, antigen presentation, and immune suppression. Karimi and colleagues [5] generated one of the first comprehensive maps of immune architecture across primary and metastatic brain tumors, revealing that immune ecosystem organization differs substantially between tumor types. Subsequent studies identified specific immune neighborhoods associated with favorable or unfavorable clinical outcomes, highlighting the potential prognostic significance of spatial immune architecture. Spatial proteomic studies further demonstrated that T-cell exclusion is not uniform throughout glioblastoma. Rather, lymphocytes often accumulate within restricted microanatomical compartments and around vessels [40] while being excluded from large portions of the tumor parenchyma. Understanding the mechanisms underlying these exclusion patterns may prove critical for improving immunotherapy responses in glioblastoma.

### 5.6. Treatment-Induced Ecosystem Remodeling

The final major insight emerging from spatial omics studies is that therapy reshapes entire ecosystems rather than merely selecting resistant tumor clones. Longitudinal analyses of paired primary and recurrent glioblastomas demonstrate that recurrence is frequently accompanied by substantial alterations in cellular composition, spatial organization, immune architecture, and microenvironmental signaling. While genetic evolution certainly contributes to disease progression, many of the most striking changes occur at the ecosystem level. Wang and colleagues [24] demonstrated that recurrent tumors frequently exhibit increased mesenchymal polarization accompanied by extensive remodeling of surrounding immune populations. Recent paired primary-recurrent spatial analyses further suggest that distinct evolutionary trajectories may exist across patients [8,14,16,22]. Some tumors progress toward highly inflammatory mesenchymal ecosystems characterized by profound immune suppression, whereas others develop more vascularized and immunologically active niches associated with improved clinical outcomes. These findings raise the possibility that ecosystem evolution can be an added value to genomic markers (MGMT, TERT, EGFR), being the current standard for glioblastoma patient stratification. Nowadays, the clinical use of spatial omics is hampered by high costs, slow turnaround times and computational/bioinformatical constraints. Collectively, longitudinal spatial studies highlight the dynamic nature of glioblastoma ecosystems and emphasize the need for future investigations that move beyond single time-point analyses toward comprehensive ecosystem tracking throughout disease progression and treatment.

## 6. Cohort Design and Tissue Selection Shape Biological Discovery

The evolution of spatial omics technologies has been accompanied by a parallel transformation in the patient cohorts and tissue resources used to study glioblastoma. Early investigations were primarily proof-of-concept studies performed on relatively small cohorts (<15 samples), with the primary objective of demonstrating the feasibility of emerging spatial profiling platforms. As technologies matured and became more accessible, cohort sizes gradually expanded through multicenter collaborations, institutional biobanks, and the integration of publicly available datasets (Table 1). This progression enabled the transition from highly focused exploratory studies toward increasingly comprehensive analyses involving dozens to hundreds of patients.

In parallel, the composition of patient cohorts evolved considerably. Initial studies predominantly examined treatment-naïve, newly diagnosed glioblastoma samples, reflecting the availability of surgical specimens and the early emphasis on defining baseline tumor architecture. More recent investigations have increasingly incorporated recurrent disease, recognizing that treatment-resistant tumors represent the clinically most relevant stage of disease progression. Several landmark studies have now analyzed matched primary and recurrent tumors from the same patients [6,8,14,16,19,22], enabling direct investigation of ecosystem remodeling during disease evolution and therapeutic intervention. These longitudinal cohort designs have provided unique insights into the dynamic nature of glioblastoma biology and have begun to reveal how malignant, immune, and stromal populations adapt over the course of treatment.

The increasing scale of spatial studies has also been accompanied by progressively richer clinical annotation. While early studies often focused primarily on biological discovery, more recent investigations have integrated detailed clinical information, including treatment history, survival outcomes, molecular biomarkers, radiological characteristics, and pathological features. In particular, the incorporation of MGMT promoter methylation status, extent of resection, and longitudinal outcome data have facilitated the exploration of associations between spatial ecosystem organization and patient prognosis, even though such information is often not incorporated in the analysis. The integration of spatial profiling with clinical metadata has therefore become an increasingly important component of contemporary glioblastoma research.

The biological material available for spatial profiling has likewise diversified substantially. Fresh-frozen specimens initially formed the foundation of many transcriptomic studies because of their excellent preservation of nucleic acids and compatibility with genome-wide sequencing approaches, including single-cell and spatial transcriptomics. These samples enabled the generation of the first high-resolution molecular atlases of glioblastoma and provided unprecedented insight into tumor cell states and microenvironmental interactions. At the same time, advances in assay development have progressively expanded the range of spatial analyses that can be performed on formalin-fixed paraffin-embedded (FFPE) tissue. Given that FFPE material represents the predominant form of archived clinical specimens worldwide, this development has unlocked access to extensive retrospective cohorts linked to long-term clinical follow-up. The increasing compatibility of transcriptomic and proteomic technologies with FFPE tissue has therefore greatly broadened the scope of spatial glioblastoma research.

Alongside these developments, spatial studies have adopted a variety of tissue sampling strategies. Whole-tissue sections have been extensively used to preserve the architectural complexity of glioblastoma, enabling visualization of spatial gradients extending across tumor cores, invasive margins, vascular niches, and necrotic regions. These approaches have been instrumental in revealing the spatial organization of malignant and non-malignant cell populations across the tumor ecosystem. At the same time, the construction of tissue microarrays (TMAs) has facilitated the analysis of increasingly large patient cohorts by enabling the simultaneous profiling of hundreds of tissue cores. Together, whole-section analyses and TMA-based studies have provided complementary perspectives on glioblastoma biology, balancing detailed spatial context with large-scale population-level investigation.

Collectively, these developments have transformed the landscape of spatial glioblastoma research. The field has progressed from small exploratory studies performed on limited numbers of fresh-frozen specimens toward large, clinically annotated cohorts incorporating both newly diagnosed and recurrent disease, analyzed using a diverse range of spatial transcriptomic, proteomic, and multiomic technologies. This expansion in cohort size, clinical depth, and tissue accessibility has laid the foundation for increasingly comprehensive investigations into the ecological organization and evolutionary dynamics of glioblastoma.

## 7. Persistent Limitations of Current Studies

Despite the remarkable advances enabled by spatial omics technologies, several important limitations continue to constrain their translational impact in glioblastoma research (Table 1). One of the most prominent challenges is the limited size of most spatial cohorts. The majority of discovery studies profile only a small number of tumors (<15 samples per platform), which restricts statistical power and limits the generalizability of reported findings. Even larger atlas studies frequently analyze selected subsets of available samples rather than truly representative cohorts, introducing the risk of selection bias. A second limitation is the continued underrepresentation of recurrent disease. Although recurrence is the clinically relevant stage responsible for almost all glioblastoma-related mortality, relatively few spatial studies include recurrent tumors, and only a minority investigate matched primary-recurrent pairs. The inherently static nature of spatial profiling fails to capture temporal dynamics. Consequently, it remains difficult to distinguish pre-existing spatial heterogeneity from treatment-induced adaptations, leaving the evolutionary trajectories of tumor ecosystems during therapy incompletely understood. Sampling bias represents an additional challenge. Most studies preferentially profile viable tumor regions with high cellularity and optimal tissue quality, while invasive margins, hypoxic and necrotic transition zones, perivascular niches, and adjacent brain tissue are frequently underrepresented. Given the substantial biological differences that exist between these compartments, such sampling strategies may provide an incomplete view of the tumor ecosystem, even though technical barriers may be present to reliably investigate necrotic regions which are often of poor quality. Similar biases can be introduced at the molecular level through the use of predefined gene or protein panels in targeted spatial transcriptomic and proteomic approaches. Although these targeted methods have generated important biological insights, they may overlook unexpected cellular programs and signaling pathways. Likewise, tissue microarray-based studies, while enabling cost-effective large-scale screening, sacrifice much of the regional architectural information that underlies spatial biology.

Technical and analytical heterogeneity further complicates the field. Considerable variation exists in tissue handling procedures, sample quality control, segmentation strategies, cell-type annotation methods, neighborhood definitions, and spatial statistical frameworks. Some platforms have a coarse spatial resolution, often leading to signal mixing of neighboring cells. Proper cell segmentation unfortunately remains a crucial technical flaw specific to brain tissue analysis, rendering standard nuclear-based segmentation algorithms often of insufficient quality [41]. As a result, signals of multiple molecules are being mapped onto spatial spots, bins, or pixels; inherently precluding single-molecule analysis. Furthermore, spatial or physical proximity alone does not necessarily imply functional cell–cell interactions. Therefore, spatial information should be integrated with ligand-receptor expression and additional functional testing (e.g., direct cell–cell interactions using proximity ligation) before concluding true cell–cell communication [42,43,44].

In addition, reporting of critical technical parameters, including failed samples, ROI selection criteria, and preprocessing workflows, remains inconsistent across studies [13]. Together, these factors hamper reproducibility, limit cross-study comparisons, and complicate efforts to perform robust meta-analyses. External validation cohorts, which are essential for demonstrating generalizability across independent patient populations and reducing the risk of overfitting, remain uncommon despite their recognized importance.

Perhaps most importantly, many spatial omics studies remain only loosely connected to clinical and mechanistic investigation. Clinical annotation is frequently incomplete, with inconsistent reporting of treatment regimens and response to treatment, MGMT methylation status, extent of resection, tumor location, radiological characteristics, and long-term outcomes. Observed heterogeneity in spatial omics techniques may not only capture and illustrate the underlying intrinsic biology but may be prone to treatment-related confounders (e.g., corticosteroid or chemotherapy exposure), highlighting the entanglement between treatment effects and intrinsic tumor biology [45,46]. As a result, relatively few studies have been able to directly link spatial ecosystem features to clinically actionable patient stratification strategies. To date, no spatial biomarker has entered routine clinical decision-making in glioblastoma, despite the identification of numerous candidate cellular states, niches, and interaction networks. Furthermore, many biological associations identified through spatial profiling have not yet been subjected to sufficient functional validation to establish causality or therapeutic relevance. Consequently, a substantial proportion of current spatial omics findings remain descriptive rather than mechanistically resolved.

Finally, although spatial multiomics is rapidly emerging, comprehensive integration of transcriptomic, proteomic, genomic, epigenomic, imaging, and clinical data remains relatively uncommon. Most studies continue to examine individual molecular layers in isolation, limiting the ability to reconstruct the complex regulatory networks that govern tumor progression and treatment response. Future progress will therefore depend not only on generating increasingly detailed spatial datasets, but also on integrating these data with functional experimental models, longitudinal patient cohorts, and deeply annotated clinical datasets. Such efforts will be essential to translate spatial discoveries from descriptive ecosystem maps into biologically validated and clinically actionable therapeutic strategies.

## 8. Best Practices and Roadmap Toward Standardized Spatial Omics in Glioblastoma

To accelerate clinical translation, the glioblastoma research community should move beyond individual proof-of-concept studies and adopt harmonized experimental, computational, and reporting frameworks. Rather than developing new standards from scratch, many recommendations can build upon successful initiatives established in the broader spatial omics and single-cell communities.

*Standardize tissue sampling through consensus protocols:* Spatial heterogeneity is one of the defining characteristics of GBM, yet sampling strategies differ substantially between studies. An international working group could establish consensus recommendations defining a minimum sampling framework, including collection of the tumor core, infiltrative margin, necrotic regions, and where feasible, paired primary and recurrent specimens. Such harmonized sampling approaches are already being implemented by the GBM-Space consortium [47] which performs systematic multi-region sampling combined with matched single-cell and spatial profiling across multiple tumor regions.

*Create community reference datasets:* Similar to the Human Cell Atlas and HuBMAP [48,49] initiatives, the GBM field would benefit from a publicly accessible reference atlas containing standardized spatial transcriptomic, spatial proteomic, histopathological, genomic, and clinical datasets generated across multiple institutions. These datasets should include expert-curated annotations of malignant cell states, immune populations, vascular niches, and treatment status, allowing new technologies and computational methods to be benchmarked against common reference standards. Large atlas initiatives have demonstrated that uniform processing pipelines, centralized quality control, and interactive public data portals substantially improve reproducibility and data reuse.

*Develop benchmarked bioinformatic pipelines:* Rather than relying on laboratory-specific workflows, the community should establish modular reference pipelines covering quality control, image registration, cell segmentation, normalization, batch correction, cell-type annotation, spatial domain identification, neighborhood analysis, ligand-receptor inference, and multimodal integration. These workflows should be openly available through GitHub, distributed as containerized Docker or Singularity environments, and version-controlled to ensure computational reproducibility. Benchmarking efforts similar to those initiated by the SpaceTx Consortium [50] could be adopted for GBM, allowing different computational approaches to be objectively compared using identical reference datasets.

*Establish standardized reporting guidelines:* The field would benefit from a reporting framework analogous to MIAME for microarrays or the reporting standards developed by the Human Cell Atlas. A Minimum Information for Spatial Omics Experiments (MISOE) guideline in line with common practice [51] could define the minimum metadata required for publication, including patient demographics, molecular subtype, treatment history, sampling strategy, technology platform, quality-control metrics, computational methods, software versions, and deposition of raw and processed data in publicly accessible repositories. Such standardized metadata would greatly facilitate reproducibility, cross-study comparisons, and future meta-analyses.

*Promote multimodal and cross-platform validation:* Biological discoveries should not rely on a single spatial technology. Candidate biomarkers identified by whole-transcriptome spatial profiling should routinely be validated using orthogonal approaches, including multiplex immunohistochemistry, RNAscope, or targeted imaging-based spatial transcriptomics, and subsequently reproduced in independent patient cohorts. Increasingly, integrated spatial atlases demonstrate the value of combining sequencing-based spatial transcriptomics with imaging-based approaches and single-cell datasets to generate more robust biological insights [14,15,19].

*Design studies for clinical implementation from the outset:* Discovery studies should incorporate predefined validation strategies and clearly define a translational pathway toward clinically deployable assays. Rather than focusing solely on increasingly complex molecular maps, future efforts should prioritize reducing high-dimensional signatures into robust biomarker panels that can be implemented using technologies compatible with routine pathology laboratories [13]. Prospective multicenter validation, early engagement with regulatory agencies, and collaboration with industry partners should become integral components of study design rather than downstream considerations.

Overall, these recommendations provide a realistic roadmap for the next generation of glioblastoma spatial omics studies. The field has already demonstrated that glioblastoma is organized into reproducible spatial ecosystems and that therapeutic adaptation largely reflects ecosystem remodeling rather than purely genetic evolution. The next challenge is therefore to establish shared standards for study design, computational analysis, data sharing, and biomarker validation. By leveraging successful frameworks established by initiatives such as the Human Cell Atlas, HuBMAP, SpaceTx, and GBM-Space, the GBM community can accelerate the development of reproducible spatial biomarkers that are ultimately suitable for prospective clinical trials and precision neuro-oncology.

## Figures and Tables

**Table 1:** Overview of spatial omics technologies that have been applied to glioblastoma research, examining how these platforms have been used to interrogate tumor biology, how patient cohorts and tissue specimens have been selected and characterized, and to what extent spatial datasets have been integrated with clinical and molecular information.

	Glioblastoma Patient Cohort			Clinical Characteristics	Genome Sequencing	MGMTp Methylation	Tissue Samples		Tissue Microarray	Immunehistochemistry/Immunefluorescence		Single Cell Transcriptomics		Single Cell Proteomics		Spatial/Neighbourhood Analysis
	Newly Diagnosed (*n*)	Recurrent (*n*)	Paired Samples (Y/N)	Demographics Treatment Survival Tumor Location	Technology	Available (Y/N) + Method	Paired (Y/N)	Total Amount (*n*)	Available (Y/N)	Antibodies (*n*)	Multiplex (Y/N)	Cells (*n*)	Genes (*n*)	Cells (*n*)	Proteins (*n*)	Available (Y/N)
Vanmechelen et al. BioRxiv 2026 [14]	96 (*n* = 52 discovery cohort; *n* = 44 validation cohort)	96	Y	Demographics Treatment Survival Tumor Location	TSO500 22/52 (bulk) WES 19/52 (bulk)	Y (Methylation specific PCR)	Y	111 (334 cores ND, 360 cores REC)	Y	38	Y	9095	1008	4,788,453	38	Y
Piyadasa et al. Cancer Cell 2026 [15]	82/310	21/310	N	Demographics Treatment Survival	N	N	N	630	Y	52	Y	1,288,012	11.162	1,288,012	52	Y
Spitzer et al. Nat Gen 2025 [8] Nomura et al. Nat Gen 2025 [16]	59	59	Y	Demographics Treatment Survival Tumor Location	WES 46/59 WGS 13/59 snRNA 59/59	Y	Y	121	N	na	N	246,408 malignant cells 182,897 non-malignant cells	3000	na	na	N
Ruiz-Moreno et al. Neuro-Oncology 2025 [17]	109/240	na	N	N	scRNA 109/240	N	N	13	N	na	N	988.901	209	na	na	Y
Migliozzi et al. Cancer Cell 2025 [18]	15	1	N	Demographics Tumor Location	snRNA 59/59	Y	N	16; data reuse from same consortium	N	53	Y	Dataset 1 (8 patients): CosMx 1K: 345,143 Dataset 2 (8 patients): CosMx 6K: 2,750,202 Greenwald cohort [7]: 564,944	Dataset 1: 1000 Dataset 2: 6000 Greenwald cohort [7]: 10× genomics Visium whole transcriptome	152.166	53	Y
Kim et al. Cancer Cell 2024 [19]	123	123	Y	Demographics Survival	WES 122/123 scRNA 8/246	60/123	Y	na	N	na	N	4228 malignant cells 12,258 non-malignant cells	Whole transcriptome	10,533 proteome 13,328 phospho-proteome	na	N
Greenwald et al. Cell 2024 [7]	26 (13 samples from Ravi et al. [20])	na	N	Demographics Tumor Location	N	Y	N	na; data reuse from same consortium (Ravi et al. 2022 [20])	Y	40	Y	564.944	10× genomics Visium whole transcriptome	70,618 spots (median of 8 cells/spot)	40	Y
Hoogstrate et al. Cancer Cell 2023 [6]	294 (165 G-SAM [21] + 129 GLASS [22])	na	209/294 (122 G-SAM [21] + 87 GLASS [22])	Treatment Survival	scRNA 287/294 snRNA 216/294	Y	Y	503 = 287 (G-SAM [21]) + 216 (GLASS [22]); data reuse from G-SAM [21] (EORTC)	N	7	Y	na	7425	mIHC on 15 paired samples *n* = 5032 image tiles	7	Y
Karimi et al. Nature 2023 [5]	123/139	13/139	N	Demographics	N	Y	N	389	Y	36	Y	na	na	Imaging mass cytometry 1,163,362	36	Y
Ravi et al. Cancer Cell 2022 [20]	20	na	N	Demographics Tumor Location	scRNA	Y (HumanMethylation450 (HM-450K) BeadChip)	N	28	N	39	Y	88.793	Array–based stRNA–seq (Visium 10×)	Imaging mass cytometry 82,179	39	Y
Xiao et al. Front Immunol 2022 [23]	7	na	N	Demographics	scRNA	Y	N	7	N	2	N	28.279	25,467 (1000–2000 median)	na	na	N
Wang et al. Nat Cancer 2022 [24]	49	49	36/49	Demographics Treatment Survival Tumor Location	WES 11/49 scRNA * snRNA 86	N	Y	111 = 53 newly diagnosed + 58 recurrent); data re-use of Neftel et al. [25] + Couturier et al. [26] dataset	N	17	Y	78,415 snRNA-seq 22,214 scATAC-seq 254,288 transcriptomes	Nanostring GeoMx1800	Spatial proteomics 6 samples (3 pairs)	17	Y
Varn et al. Cell 2022 [22]	128	128	Y	Demographics Survival Tumor Location	WES WGS scRNA	N	Y	256	N	6	Y	55,284 (11 patients)	4132; not spatial	na	6	N
Pombo Antunes et al. Nature Neurosci 2021 [27]	7	4	N	Demographics Treatment	scRNA	N	N	11	N	3	Y	64.173	ScRNAseq (whole transcriptome); CITE-seq	14.793	12	N
Couturier et al. Nat Comm 2020 [26]	16	N	N	Tumor Location	scRNA	N	N	16	N	7	N	53.586	2000–5000; not spatial	42.983	9	N
Neftel et al. Cell 2019 [25]	20	N	N	Demographics Tumor Location	WES scRNA	N	N	20	N	na	N	5742	RNA in sity hybridisation ~5000	N	N	N
Wang et al. Cancer Disc 2019 [28]	22	N	N	Demographics	WES scRNA snRNA	N	N	22	N	4	N	31.281	ScRNAseq (whole transcriptome); not spatial	N	N	N

Legend: CITE-seq: Cellular Indexing of Transcriptomes and Epitopes by Sequencing; CosMx: CosMx Spatial Molecular Imager; EORTC: European Organisation for Research and Treatment of Cancer; GLASS: The Glioma Longitudinal AnalySiS (GLASS) consortium; G-SAM: GMM-based segment anything model; GeoMx: GeoMx Digital SPatial Profiler (DSP); MGMT: O^6^-methylguanine-DNA methyltransferase; mIHC: Multiplex immunohistochemistry; N: no; na: not applicable; ND: newly diagnosed; PCR: Polymerase Chain Reaction; REC: recurrent; ROI: region of interest; scATACseq: Single-Cell Assay for Transposase-Accessible Chromatin using sequencing; scRNAseq: Single-Cell RNA-sequencing; snRNAseq: Single-Nucleus RNA Sequencing; stRNAseq: Spatial Transcriptome RNA sequencing; TSO500: TruSight Oncology 500; WES: whole exome sequencing; WGS: whole genome sequencing; WHO: World Health Organization; wt: wild-type; Y: yes.

**Table 2:** Glossary with an overview of the features of the most important spatial omics platforms in glioblastoma research. The table lists the respective techniques, including the principles of detection, target type, spatial resolution, plexity, and tissue type compatibility, as well as their strengths and weaknesses.

Technology	Detection Principle	Target Type	Discovery Or Targeted	Spatial Resolution	Plexity	Tissue Compatibility	Main Strengths	Main Limitations
Visium and Visium HD (10× Genomics)	Spatial barcoded capture spots	Whole transcriptome RNA	Discovery	~55 µm spot; 2–8 µm (for the HD version)	~18,000+ genes	Fresh frozen (FFPE targeted assay)	Transcriptome-wide profiling; broad adoption	Limited single-cell resolution
Xenium (10× Genomics)	In situ hybridization + imaging	RNA	Targeted	Subcellular	Up to ~5000 genes	FFPE & fresh frozen	High sensitivity; single-cell segmentation	Limited sensitivity
CosMx SMI (Bruker)	Single-molecule imaging	RNA ± protein	Targeted	Subcellular	From 1000-~18,000+ genes	FFPE & fresh frozen	Very high plexity	Long acquisition times
MERFISH/MERSCOPE (Vizgen)	Sequential FISH	RNA	Targeted	Subcellular	1000–10,000 genes	Mainly fresh frozen	Extremely sensitive	Specialized instrumentation
GeoMx DSP (Bruker)	UV photocleavage indexed probes	RNA & protein	Targeted	ROI-based (10–600 µm)	~18,000 RNA or >100 proteins	FFPE	Flexible ROI selection	No single-cell resolution
RNAscope (ACD)	Chromogenic/fluorescent ISH	RNA	Highly targeted	Single molecule	1–12+ genes	FFPE	Excellent sensitivity	Low multiplexing
PhenoCycler/CODEX (Quanterix)	DNA-barcoded antibodies	Protein	Targeted	Single-cell	1–100+ proteins	FFPE & fresh frozen	Highly multiplexed proteins	Antibody labeling is labor intensive
COMET (Biotechne)	Sequential immunofluorescence	RNA ± protein	Targeted	Single-cell	1–60+ proteins	FFPE & fresh frozen	Automated; rapid	Is combined with RNAscope in same run
MILAN mIHC; cycIF (academic)	Iterative stain/strip	Protein	Targeted	Single-cell	1–60+ proteins	FFPE	Flexible, cost-effective	Labor-intensive but scalable
MACSima (Miltenyi)	Automated cyclic IF	RNA ± protein	Targeted	Single-cell	100+ proteins	FFPE & fresh frozen	Very high protein plexity	Long imaging runs
Hyperion MIBI-TOF (Standard Biotools)	Metal-tagged antibodies	Protein	Targeted	~200 nm–10 µm	40–50 proteins	FFPE & fresh frozen	Quantitative; no spectral overlap	Slow; tissue destroyed

Legend: CODEX: Co-Detection by indexing; COMET: CosMx: CosMx Spatial Molecular Imager; cycIF: Cyclic Immunofluorescence; DNA: deoxyribonucleic acid; FFPE: Formalin-fixed paraffin-embedded; FISH: Fluorescence in situ hybridization; GeoMx: GeoMx Digital SPatial Profiler (DSP); IF: Immunofluorescence; ISH: In situ Hybridization; MERFISH: Multiplexed Error-Robust Fluorescence in situ Hybridization; MIBI-TOF: Multiplexed Ion Beam Imaging by Time of Flight; mIHC: Multiplex Immunohistochemistry; MILAN: Multiple iterative labeling by antibody neodeposition; μm: micrometer; RNA: ribonucleic acid; ROI: Region of interest; UV: Ultraviolet.

## Data Availability

No new data were created or analyzed in this study. Data sharing is not applicable to this article.

## Abbreviations

The following abbreviations are used in this manuscript:

AC-like	Astrocyte-like
CL-like	Cilia-like
FFPE	Formalin-fixed paraffin-embedded
GBM	Glioblastoma
GPC-like	Glial progenitor cell-like
MES-like	Mesenchymal-like
MIAME	Minimum information about a microarray experiment
MISOE	Minimum Information for Spatial Omics Experiments
NL-like	Neuron-like
NPC-like	Neural progenitor-like
OPC-like	Oligodendrocyte progenitor-like
PDGF	Platelet derived growth factor
ROI	Region of interest
TMA	Tissue microarrays
TME	Tumor microenvironment
VEGF	Vascular endothelial growth factor
